# Novel erythropoiesis stimulating protein (NESP) for the treatment of anaemia of chronic disease associated with cancer

**DOI:** 10.1054/bjoc.2001.1749

**Published:** 2001-04

**Authors:** R E Smith, I A Jaiyesimi, L A Meza, N S Tchekmedyian, D Chan, H Griffith, S Brosman, R Bukowski, M Murdoch, M Rarick, A Saven, A B Colowick, A Fleishman, U Gayko, J Glaspy

**Affiliations:** South Carolina Oncology Associates, 1301 Taylor, Suite IA, Columbia, SC 29201, USA; William Beaumont Hospital, 3577 West 13 Mile Road, Suite 40 Royal Oak, MI 48073, USA;; Southwest Oncology Associates, 443 Heymann Blvd, Suite A, Lafayette, LA 70503, USA;; Pacific Shores Medical Group Inc, 1043 Elm Ave, Suite 104 Long Beach, CA 90813-3244, USA; Cancer Care Associates Medical Group, 514 North Prospect St, Redondo Beach, CA 90277-3026, USA; Memorial Clinic Research Center 520 Lilly Road,NE Building 3, Olympia, WA 98506, USA; Pacific Clinical Research, 1260 15^th^Street, Suite 1200, Santa Monica, CA 90404, USA; The Cleveland Clinic Foundation, Desk S33, 9500 Euclid Avenue, Cleveland, OH 44195, USA; Urology Associates, 7500 Hanover Parkway, Suite 206, Greenbelt, MD 20770, USA; Kaiser Permanente Northwest Division, 3600 North Interstate, Portland, OR 97227, USA; Scripps Clinic, 10666 North Torrey Pines Road La Jolla, CA 92037, USA; Amgen Inc, One Amgen Center Drive, Thousand Oaks, CA 91320-1799, USA; Amgen Inc, One Amgen Center Drive, M/S 24-1-C, Thousand Oaks, CA 91320-1799, USA; Amgen Inc, One Amgen Center Drive; M/S 24-1-C, Thousand Oaks, CA 91320-1799, USA; UCLA School of Medicine, 200 Medical Plaza, Suite 120, Los Angeles, CA 90024, USA

**Keywords:** anaemia, cancer, chemotherapy, chronic disease, darbepoetin alfa, erythropoietin

## Abstract

Anaemia is a common haematologic disorder in patients with cancer and has a multifactorial aetiology, including the effects of the malignancy itself and residual effects from previous therapy. Novel erythropoiesis stimulating protein (NESP, darbepoetin alfa), a protein with additional sialic acid compared with erythropoietin (EPO), stimulates erythropoiesis by the same mechanism as recombinant human erythropoietin (rHuEPO) but it is biochemically distinct. NESP, with its approximately 3-fold greater serum half-life, can maintain haemoglobin levels as effectively as rHuEPO in anaemic patients with chronic renal failure and do so with less frequent dosing. We investigated the ability of NESP to safely increase haemoglobin levels of anaemic patients with non-myeloid malignancies not receiving chemotherapy. NESP was administered under the supervision of a physician at doses of 0.5, 1.0, 2.25 or 4.5 mcg kg^−1^wk^−1^for a maximum of 12 weeks. This report includes 89 patients completing the study by November 2000. NESP was well tolerated, with no reported dose-limiting toxicities or treatment-related severe adverse events. Increasing doses of NESP corresponded with increased efficacy. The percentage (95% confidence interval) of patients responding ranged from 61% (42%, 77%) in the 1.0 mcg kg^−1^wk^−1^group to 83% (65%, 94%) in the 4.5 mcg kg^−1^wk^−1^group. © 2001 Cance Cancer Research Campaign


*British Journal of Cancer* (2001) **84** (Supplement 1), 24–30
© 2001 Cancer Research Campaign
doi: 10.1054/ bjoc.2001.1749, available online at http://www.idealibrary.com on IDEAL®

### Novel erythropoiesis stimulating protein (NESP) for the treatment of anaemia of chronic disease associated with cancer

**RE Smith, Jr[1], IA Jaiyesimi[2], LA Meza[3], NS Tchekmedyian[4], D Chan[5], H Griffith[6], S Brosman[7], R Bukowski[8], M Murdock[9], M Rarick[10], A Saven[11], AB Colowick[12], A Fleishman[13], U Gayko[14] and J Glaspy[15]**

[1]South Carolina Oncology Associates, 1301 Taylor, Suite IA, Columbia, SC 29201, USA; [2]William Beaumont Hospital, 3577 West 13 Mile Road, Suite 404, Royal Oak, MI 48073, USA; [3]Southwest Oncology Associates, 443 Heymann Blvd, Suite A, Lafayette, LA 70503, USA; [4]Pacific Shores Medical Group Inc, 1043 Elm Ave, Suite 104, Long Beach, CA 90813-3244, USA; [5]Cancer Care Associates Medical Group, 514 North Prospect St, Redondo Beach, CA 90277-3026, USA; [6]Memorial Clinic Research Center, 520 Lilly Road, NE, Building 3, Olympia, WA 98506, USA; [7]Pacific Clinical Research, 1260 15th Street, Suite 1200, Santa Monica, CA 90404, USA; [8]The Cleveland Clinic Foundation, Desk S33, 9500 Euclid Avenue, Cleveland, OH 44195, USA; [9]Urology Associates, 7500 Hanover Parkway, Suite 206, Greenbelt, MD 20770, USA; [10]Kaiser Permanente Northwest Division, 3600 North Interstate, Portland, OR 97227, USA; [11]Scripps Clinic, 10666 North Torrey Pines Road, La Jolla, CA 92037, USA; [12]Amgen Inc, One Amgen Center Drive, Thousand Oaks, CA 91320-1799, USA; [13]Amgen Inc, One Amgen Center Drive, M/S 24-1-C, Thousand Oaks, CA 91320-1799, USA; [14]Amgen Inc., One Amgen Center Drive; M/S 24-1-C, Thousand Oaks, CA 91320-1799, USA; [15]UCLA School of Medicine, 200 Medical Plaza, Suite 120, Los Angeles, CA 90024, USA

**Summary** Anaemia is a common haematologic disorder in patients with cancer and has a multifactorial aetiology, including the effects of the malignancy itself and residual effects from previous therapy. Novel erythropoiesis stimulating protein (NESP, darbepoetin alfa), a protein with additional sialic acid compared with erythropoietin (EPO), stimulates erythropoiesis by the same mechanism as recombinant human erythropoietin (rHuEPO) but it is biochemically distinct. NESP, with its approximately 3-fold greater serum half-life, can maintain haemoglobin levels as effectively as rHuEPO in anaemic patients with chronic renal failure and do so with less frequent dosing. We investigated the ability of NESP to safely increase haemoglobin levels of anaemic patients with non-myeloid malignancies not receiving chemotherapy. NESP was administered under the supervision of a physician at doses of 0.5, 1.0, 2.25 or 4.5 mcg $kg^{-1}$ $wk^{-1}$ for a maximum of 12 weeks. This report includes 89 patients completing the study by November 2000. NESP was well tolerated, with no reported dose-limiting toxicities or treatment-related severe adverse events. Increasing doses of NESP corresponded with increased efficacy. The percentage (95% confidence interval) of patients responding ranged from 61% (42%, 77%) in the 1.0 mcg $kg^{-1}$ $wk^{-1}$ group to 83% (65%, 94%) in the 4.5 mcg $kg^{-1}$ $wk^{-1}$ group.
© 2001 Cancer Research Campaign

**Keywords**: anaemia; cancer; chemotherapy; chronic disease; darbepoetin alfa; erythropoietin

Anaemia is common in patients with cancer and contributes to the morbidity associated with this disease. Although patients with cancer may have factors such as chemotherapy, radiotherapy, haemolysis, bleeding, poor nutrition or bone marrow infiltration contributing to anaemia, for most patients, the anaemia is primarily due to the erythropoietic aberrations associated with chronic disease. The anaemia of chronic disease is associated with both a relative endogenous erythropoietin (EPO) deficiency (Miller et al, 1990; Ozguroglu et al, 2000) and a blunted erythropoietic response to EPO, probably caused by inflammatory cytokines (Jelkmann, 1998).

Anaemia is associated with numerous symptoms, including depression, impaired cognitive function, and fatigue, which is a primary complaint of patients receiving chemotherapy (Ludwig and Fritz, 1998). Treatment options for anaemia in patients with cancer not receiving chemotherapy include administration of red blood cell transfusions if the anaemia is severe, or watchful waiting if the anaemia is mild or moderate.

Several studies have examined recombinant human erythropoietin (rHuEPO) in the treatment of the chronic anaemia of cancer not associated with chemotherapy. Administration improved haematocrit or haemoglobin values after 8 to 12 weeks of treatment in patients with anaemia due to cancer in the absence of concomitant chemotherapy (Abels et al, 1991; Ludwig et al, 1994). However, these studies failed to show statistically significant reductions in the number of red blood cell transfusions needed, probably because the studies were inadequately powered to detect a benefit or because the dose and length of study were not sufficient to achieve the desired effect. In a separate study, Henry and Abels (1994) demonstrated significant decreases in transfusion requirements of anaemic patients not receiving chemotherapy after 6 months of treatment. All studies required administration of 100 to 150 U $kg^{-1}$ rHuEPO as a subcutaneous injection three times per week under the supervision of a physician. In most studies, a single dose increase was permitted for non-responding patients. The dose–response relationship for rHuEPO used in this setting has not been fully characterized.

Darbepoetin alfa is a novel erythropoiesis stimulating protein (NESP) that stimulates erythropoiesis in the same manner as rHuEPO. NESP is biochemically distinct from rHuEPO. It has additional sialic acid that has been shown to confer an increased terminal half-life in animal models, patients with chronic renal

*Correspondence to:* J Glaspy

This study was supported by Amgen Inc, Thousand Oaks, California.

failure, and cancer patients receiving multiple cycles of chemotherapy (Egrie et al, 1997; Macdougall et al, 1999; Glaspy et al, 2000). Increasing the half-life of active drug may confer a clinical advantage by allowing less frequent dosing and maintaining stable haemoglobin levels, attributes that are potentially valuable in chronic indications. Animal studies have indicated that NESP can alleviate the anaemia of chronic disease (Cooke et al, 2000), a model for one type of anaemia seen in cancer patients not receiving chemotherapy.

The purpose of this study was to assess the safety of NESP administered by subcutaneous injection one time per week to patients with chronic anaemia who were not receiving chemotherapy and to determine the clinically effective doses of NESP in this setting during a 12-week treatment phase, and to begin to characterize the dose–response relationship. Additionally, the effects of NESP on health-related quality of life were assessed using the FACT-Fatigue scales (Cella, 1997).

### PATIENTS AND METHODS

### Patients

The institutional review boards of the participating centres approved the protocol, and patients gave written informed consent before the study was started. Patients who were at least 18 years of age with non-myeloid malignancy and anaemia (haemoglobin ≤ 11.0 g $dl^{-1}$), and who were not currently receiving or planning to receive cytotoxic chemotherapy or external beam radiotherapy within 16 weeks were eligible to enrol. Other key eligibility criteria included Eastern Cooperative Oncology Group (ECOG) performance status of 0 to 2, platelet count $\geq 50 \times 10^9$ $l^{-1}$, and anaemia predominantly resulting from cancer or previous treatment with chemotherapy or radiotherapy. All patients were required to have adequate iron stores, and adequate liver and renal function. Patients were excluded from enrolment if they had a primary or metastatic malignancy involving the central nervous system, had active bleeding or haemolysis, had received chemotherapy within 8 weeks of enrolment, had received red blood cell transfusions within 16 days of enrolment, had an unstable medical condition (e.g. angina, arrhythmia, hypertension, clinically significant systemic infection or chronic inflammatory disease other than cancer), or had a primary haematologic disorder that could cause anaemia.

### Study drug

Novel erythropoiesis stimulating protein (NESP) (darbepoetin alfa, ARANESP™, Amgen Inc, Thousand Oaks, CA) is purified from cell culture medium of a NESP-expressing Chinese hamster ovary (CHO) cell line (Egrie et al, 1997). NESP was provided in a liquid formulation, containing per ml: 0.25% human serum albumin, 140 mM sodium chloride and 20 mM sodium phosphate.

### Study design

This was an open-label, sequential dose-escalation, dose-finding study for treatment of patients with chronic anaemia of cancer. NESP was administered under the supervision of a physician as a subcutaneous injection once per week (NESP 0.5, 1.0, 2.25 or 4.5 mcg $kg^{-1}$ $wk^{-1}$) commencing on study day 1 (within 3 days of study enrolment) and administered on the same day of the week throughout the 12-week dosing period. Standard tests and observations were performed throughout the dosing period (i.e. weekly complete blood counts and monthly coagulation, blood chemistry, serum iron, iron-binding capacity, ferritin and transferrin saturation). Additional monthly blood samples were collected for antibody testing. Patients were permitted to receive red blood cell transfusions as clinically indicated and transfusions were recommended if the patient's haemoglobin decreased to ≤ 8.0 g $dl^{-1}$.

The follow-up period started immediately after the last dose of study drug administration and continued for 4 weeks. The same standard tests and observations were performed during this period.

A health-related quality of life questionnaire was completed by patients every 2 weeks during the 16-week study. The questionnaire contained the FACT-G and the FACT-Anaemia scales. The FACT-Fatigue scale, reported in this paper, is a component of the FACT-Anaemia scale.

### Dose adjustments

The dosage of NESP was initially reduced to the next lowest dose level if a patient's haemoglobin increased ≥ 2.0 g $dl^{-1}$ in the previous 28-day period in the absence of red blood cell transfusion. With the implementation of a protocol amendment, NESP was reduced to the next lowest dose based on these criteria, but only if the patient's haemoglobin was above 13.0 g $dl^{-1}$. Patients in the NESP 0.5 and 1.0 mcg $kg^{-1}$ $wk^{-1}$ cohorts were enrolled before this amendment and patients in the NESP 2.25 and 4.5 mcg $kg^{-1}$ $wk^{-1}$ cohorts were enrolled after this amendment. NESP was withheld if the haemoglobin value was > 15.0 g $dl^{-1}$ for men or > 14.0 g $dl^{-1}$ for women. NESP could be reinstated at the next lowest dose level if the haemoglobin value subsequently decreased to < 13.0 g $dl^{-1}$. Only one dose reduction per patient was permitted.

### Safety endpoints

Safety was monitored on an ongoing basis during the study period and consisted of adverse event reports, concomitant medications, laboratory tests and vital signs. Serum samples were tested at monthly intervals using a standard test to detect the development, if any, of antibodies to NESP.

### Efficacy endpoints

The efficacy of NESP was assessed based on the proportion of patients who achieved a haemoglobin response, defined as a ≥ 2.0 g $dl^{-1}$ increase from the baseline haemoglobin in the absence of a red blood cell transfusion within the preceding 28 days. Haemoglobin correction, defined as a post-baseline haemoglobin value ≥ 12.0 g $dl^{-1}$ in the absence of a red blood cell transfusion within the preceding 28 days, was also measured. Furthermore, change in baseline haemoglobin compared with week 4 and the end of treatment, incidence of red blood cell transfusions from week 5 to the end of treatment, time to haemoglobin response, and time to haemoglobin correction were assessed. In addition, the feasibility, reliability and validity of the quality of life questionnaire (FACT-Fatigue) in this setting was assessed.

### Statistical analysis

All patients who received at least one dose of NESP were evaluable for the efficacy and safety analyses. Data were analysed by

© 2001 Cancer Research Campaign 

dose cohort. Descriptive statistics summarized demographic and baseline data (for categorical data: number of subjects, percentages; for continuous data: mean, standard deviation (SD), median, minimum and maximum). The mean and 95% confidence interval (based on the normal approximation) were calculated for the change in haemoglobin from baseline to week 4 and to end of treatment. If the week 4 or end of treatment visit occurred within 28 days of a red blood cell transfusion or was missing, the last haemoglobin value before this visit was used (if no red blood cell transfusion had been given within the previous 28 days). Proportions and 95% confidence intervals (based on the Clopper–Pearson method) were calculated for the incidence of haemoglobin response, haemoglobin correction, and number of red blood cell transfusions from weeks 5 through 12. Kaplan–Meier plots were generated for time (week) to haemoglobin response and haemoglobin correction.

Health-related quality of life data were analysed by haemoglobin response (< 0 g $dl^{-1}$, 0 to 2 g $dl^{-1}$ and > 2 g $dl^{-1}$ change in haemoglobin) at the end of the 12-week study.

### RESULTS

### Patient disposition and baseline characteristics

This study was conducted at 10 sites with one site encompassing a network of approximately 20 active satellite sites. One hundred and five patients were enrolled into the study; 89 had completed the study by November 2000, and their data are included in this report. Of these 89 patients, 87 (98%) received at least one dose of study drug (NESP 0.5 mcg $kg^{-1}$ $wk^{-1}$, $n$ = 6; NESP 1.0 mcg $kg^{-1}$ $wk^{-1}$, $n$ = 33; NESP 2.25 mcg $kg^{-1}$ $wk^{-1}$, $n$ = 18; NESP 4.5 mcg $kg^{-1}$ $wk^{-1}$, $n$ = 30). Two patients in the 2.25 mcg $kg^{-1}$ $wk^{-1}$ cohort did not receive NESP and are not included in the efficacy or safety analyses. Limited conclusions can be drawn from the NESP 0.5 mcg $kg^{-1}$ $wk^{-1}$ dose cohort because of the small sample size ($n$ = 6), but the data are presented for completeness. Of the 87 patients who received at least one dose of study drug, the mean (SD) age was 70.3 (12.0) years; 55% were men; the most common tumours were breast and genitourinary; most patients had an ECOG status < 2; and the mean (SD) baseline haemoglobin was 9.87 (1.01) g $dl^{-1}$ (Table 1).

Overall, 78% of the 87 patients completed the 12-week dosing regimen per protocol: 67%, 73%, 78% and 87% in the NESP 0.5, 1.0, 2.25 and 4.5 mcg $kg^{-1}$ $wk^{-1}$ cohorts, respectively. Reasons for not completing study included death (3%), adverse events (2%) and miscellaneous (1% to 4% each).

### Study drug dose adjustment

None of the patients in the NESP 0.5 mcg $kg^{-1}$ $wk^{-1}$ group, 10 (30%) in the NESP 1.0 mcg $kg^{-1}$ $wk^{-1}$ group, 3 (17%) in the NESP 2.25 mcg $kg^{-1}$ $wk^{-1}$ group and 6 (20%) in the NESP 4.5 mcg $kg^{-1}$ $wk^{-1}$ group had their dose of NESP reduced because of a rapid

**Table 1** Patient baseline demographics and clinical characteristics

| | NESP (mcg $kg^{-1}$ $wk^{-1}$) | | | | |
|---|---|---|---|---|---|
| | **0.5 ($n$ = 6)** | **1.0 ($n$ = 33)** | **2.25 ($n$ = 18)** | **4.5 ($n$ = 30)** | **ALL NESP ($n$ = 87)** |
| Age, years | | | | | |
| Mean (SD) | 53.2 (8.6) | 69.7 (13.5) | 71.1 (9.5) | 74.0 (9.1) | 70.3 (12.0) |
| Sex, $n$ (%) | | | | | |
| Female | 5 (83.3%) | 14 (42.4%) | 8 (44.4%) | 12 (40.0%) | 39 (44.8%) |
| Male | 1 (16.7%) | 19 (57.6%) | 10 (55.6%) | 18 (60.0%) | 48 (55.2%) |
| Primary site of disease, $n$ (%) | | | | | |
| Breast | 4 (66.7%) | 9 (27.3%) | 4 (22.2%) | 7 (23.3%) | 24 (27.6%) |
| Lung | | 1 (3.0%) | 2 (11.1%) | 2 (6.7%) | 5 (5.7%) |
| Gastrointestinal | | 2 (6.1%) | 2 (11.1%) | 4 (13.3%) | 8 (9.2%) |
| Gynaecologic | | 1 (3.0%) | | 1 (3.3%) | 2 (2.3%) |
| Genitourinary | 1 (16.7%) | 11 (33.3%) | 4 (22.2%) | 12 (40.0%) | 28 (32.2%) |
| Other solid tumour | 1 (16.7%) | 3 (9.1%) | | 1 (3.3%) | 5 (5.7%) |
| Lymphoid malignancy | | 6(18.2%) | 6 (33.3%) | 3 (10.0%) | 15 (17.2%) |
| ECOG, $n$ (%) | | | | | |
| 0 | 2 (33.3%) | 11 (33.3%) | 8 (44.4%) | 7 (23.3%) | 28 (32.2%) |
| 1 | 3 (50.0%) | 17 (51.5%) | 8 (44.4%) | 18 (60.0%) | 46 (52.9%) |
| 2 | 1 (16.7%) | 5 (15.2%) | 2 (11.1%) | 5 (16.7%) | 13 (14.9%) |
| Hgb, g $dl^{-1}$ | | | | | |
| Mean (SD) | 9.60 (1.53) | 9.80 (0.91) | 9.80 (1.14) | 10.05 (0.94) | 9.87 (1.01) |
| Median | 9.65 | 9.90 | 10.05 | 10.45 | 10.10 |
| Min, Max | 7.4, 11.4 | 7.3, 11.0 | 7.4, 11.2 | 7.8, 11.4 | 7.3, 11.4 |
| Endogenous EPO, mU $ml^{-1}$ | | | | | |
| Mean (SD) | 65.71 (80.41) | 45.68 (42.48) | 56.48 (49.04) | 41.76 (56.77) | 48.30 (50.65) |
| Median | 29.73 | 31.05 | 36.79 | 26.01 | 29.90 |
| Min, Max | 12.0, 206.6 | 12.0, 180.7 | 12.0, 163.9 | 12.0, 278.5 | 12.0, 278.5 |

ECOG = Eastern Cooperative Oncology Group performance status; EPO = erythropoietin; Hgb = haemoglobin; SD = standard deviation.

© 2001 Cancer Research Campaign increase in haemoglobin. The higher dose reduction rate in the NESP 1.0 mcg $kg^{-1}$ $wk^{-1}$ group compared with either the rate in the NESP 2.25 or the NESP 4.5 mcg $kg^{-1}$ $wk^{-1}$ group can probably be explained by the fact that the latter 2 cohorts were enrolled after the amendments to the protocol re-defined dose reduction in response to rapid increases in haemoglobin. None of the patients in either the NESP 0.5 or the NESP 1.0 mcg $kg^{-1}$ $wk^{-1}$ groups, 6 (33%) patients in the NESP 2.25 mcg $kg^{-1}$ $wk^{-1}$ group, and 11 (37%) patients in the NESP 4.5 mcg $kg^{-1}$ $wk^{-1}$ group had their dose of study drug withheld because their haemoglobin values were > 14.0 g $dl^{-1}$ (women) or > 15.0 g $dl^{-1}$ (men).

### Safety results

At the time of this report, 87 patients were evaluable for safety. Eighty-six (99%) patients reported at least one adverse event. The most frequently reported adverse events were fatigue, asthenia, fever, peripheral oedema, pain and influenza-like symptoms. Nine (10%) of the adverse events were reported as treatment-related. Twenty patients (23%) experienced serious adverse events that were reported by the investigator as not related to treatment. Seven (8%) of the adverse events resulted in discontinuation of study; none of these events were reported as related to treatment. Five (6%) patients died while on study, and all deaths were attributed to disease progression and reported as not related to treatment. Aside from the expected increase in erythropoietic elements, laboratory findings were consistent with the population studied. All patients who received study drug had at least one post-baseline sample tested for antibodies to NESP. None of these samples were reactive, indicating that no antibodies to NESP had been detected. No unexpected trends were seen that suggested a drug-related adverse effect and no dose-related relationships were apparent for reported adverse events. No clinically significant changes in vital signs were noted for any cohort.

### Efficacy results

Because of the small sample size ($n$ = 6), the NESP 0.5 mcg $kg^{-1}$ $wk^{-1}$ cohort is not discussed, but the data are presented in figures and tables.

Figure 1 demonstrates a dose-dependent relationship between NESP and the proportion of patients achieving a haemoglobin response and haemoglobin correction. In panel A, the percentage of patients who achieved a haemoglobin response is plotted for each cohort. Patients who received at least one dose of study drug had a response rate (95% confidence interval) of 61% (42%, 77%), 67% (41%, 87%) and 83% (65%, 94%) in the NESP 1.0, 2.25 and 4.5 mcg $kg^{-1}$ $wk^{-1}$ groups, respectively. Panel B of Figure 1 shows the percentage of patients who achieved a haemoglobin correction. The correction rate (95% confidence interval) for patients who received at least one dose of NESP was 61% (42%, 77%), 72% (47%, 90%), and 80% (61%, 92%) in the NESP 1.0, 2.25 and 4.5 mcg $kg^{-1}$ $wk^{-1}$ cohorts, respectively, suggesting a dose–response relationship. All doses studied met the predefined criteria for clinical efficacy (i.e. at least 50% of patients achieving a 2 g $dl^{-1}$ increase in haemoglobin over baseline).

The analysis of change from baseline haemoglobin also suggests a dose–response relationship (Table 2). The mean change at week 4 (95% confidence interval) for patients receiving at least one dose of study drug was 0.68 (0.22, 1.14), 0.84 (0.22, 1.46) and 1.26 (0.85, 1.67) in the NESP 1.0, 2.25 and 4.5 mcg $kg^{-1}$ $wk^{-1}$ cohorts, respectively. By the end of treatment, the mean change

**Table 2** Change from baseline haemoglobin and red blood cell transfusion

| | NESP (mcg $kg^{-1}$ $wk^{-1}$) | | | |
|---|---|---|---|---|
| | **0.5** | **1.0** | **2.25** | **4.5** |
| Change from baseline haemoglobin at week 4, g $dl^{-1}$ | | | | |
| Mean (95% CI) | –0.10 (–1.08, 0.88) | 0.68 (0.22, 1.14) | 0.84 (0.22, 1.46) | 1.26 (0.85, 1.67) |
| Change from baseline haemoglobin at the end of treatment, g $dl^{-1}$ | | | | |
| Mean (95% CI) | 1.45 (–0.52, 3.42) | 1.71 (0.91, 2.52) | 2.63 (1.54, 3.71) | 2.91 (2.17, 3.65) |
| RBC transfusions from week 5 to the end of treatment | | | | |
| Number of patients ($n$) | 1 | 8 | 3 | 2 |
| Percent of patients (95% CI) | 17 (0, 64) | 24 (11, 42) | 17 (4, 41) | 7 (1, 22) |

CI = confidence interval.
Note: Post-baseline haemoglobin values that occurred within 28 days of a red blood cell transfusion were excluded in calculating the mean. The last value was carried forward for patients without a valid week 4 or end of treatment haemoglobin value.

**Table 3** Health-related quality of life change from baseline to week 12 by haemoglobin change

| HRQOL SCORE | Hgb < 0 g $dl^{-1}$ | Hgb 0 to 2 g $dl^{-1}$ | Hgb > 2 g $dl^{-1}$ |
|---|---|---|---|
| FACT-Fatigue | | | |
| Mean | –5.2 | 6.9 | 9.0 |
| Median | –2.5 | 4.5 | 7.0 |
| SD | 10.7 | 9.7 | 10.8 |
| Quartiles | –8.0, 0.0 | 1.0, 11.0 | 2.0, 15.0 |
| Range | –27.0, 10.0 | –3.0, 31.0 | –10.0, 36.0 |
| *n* | 10 | 10 | 46 |

HRQOL = health-related quality of life; Hgb = haemoglobin.

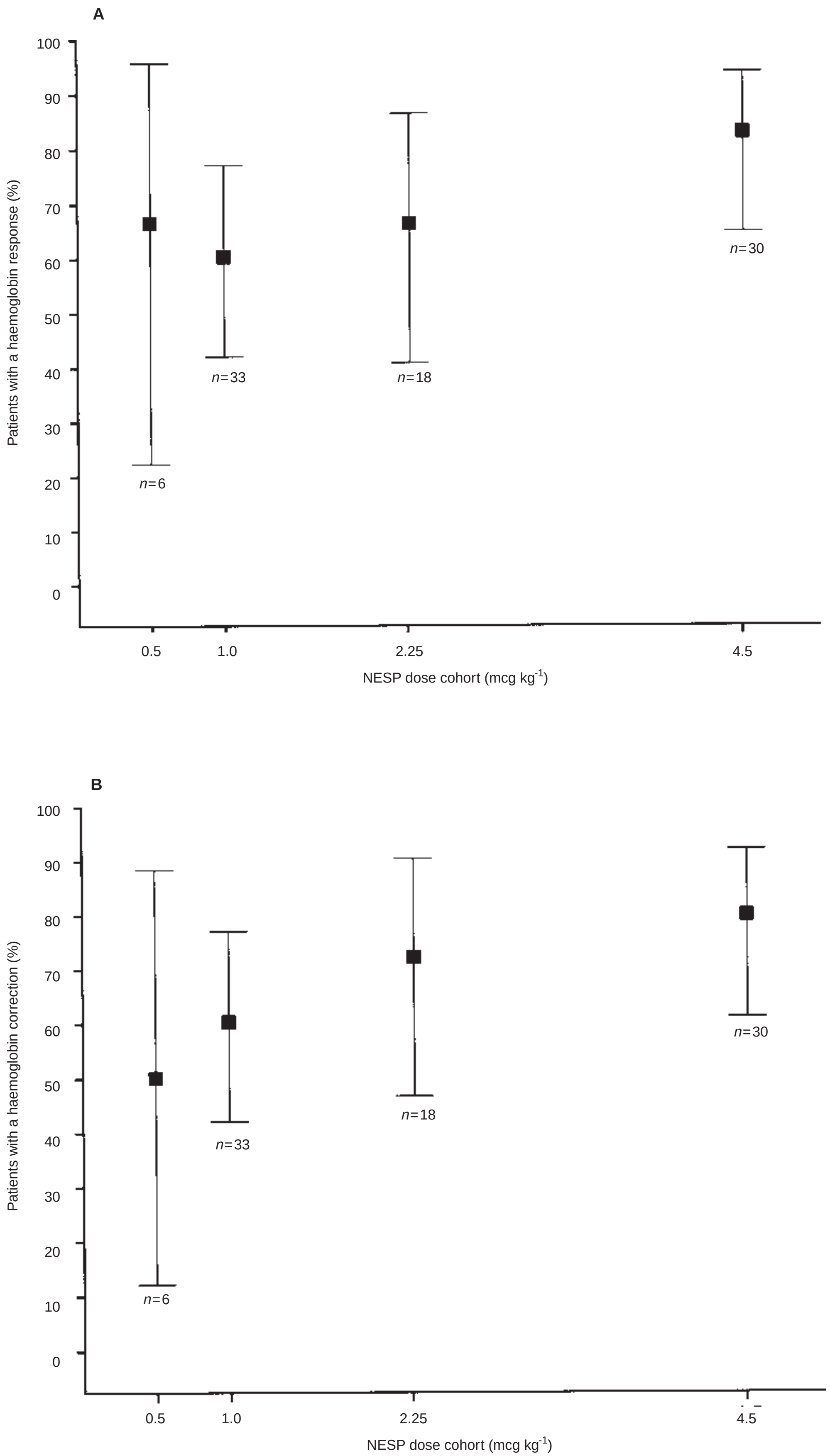

**Figure 1** The percentage of patients with a haemoglobin response (panel **A**) and haemoglobin correction (panel **B**) with exact 95% confidence intervals

*© 2001 Cancer Research Campaign*

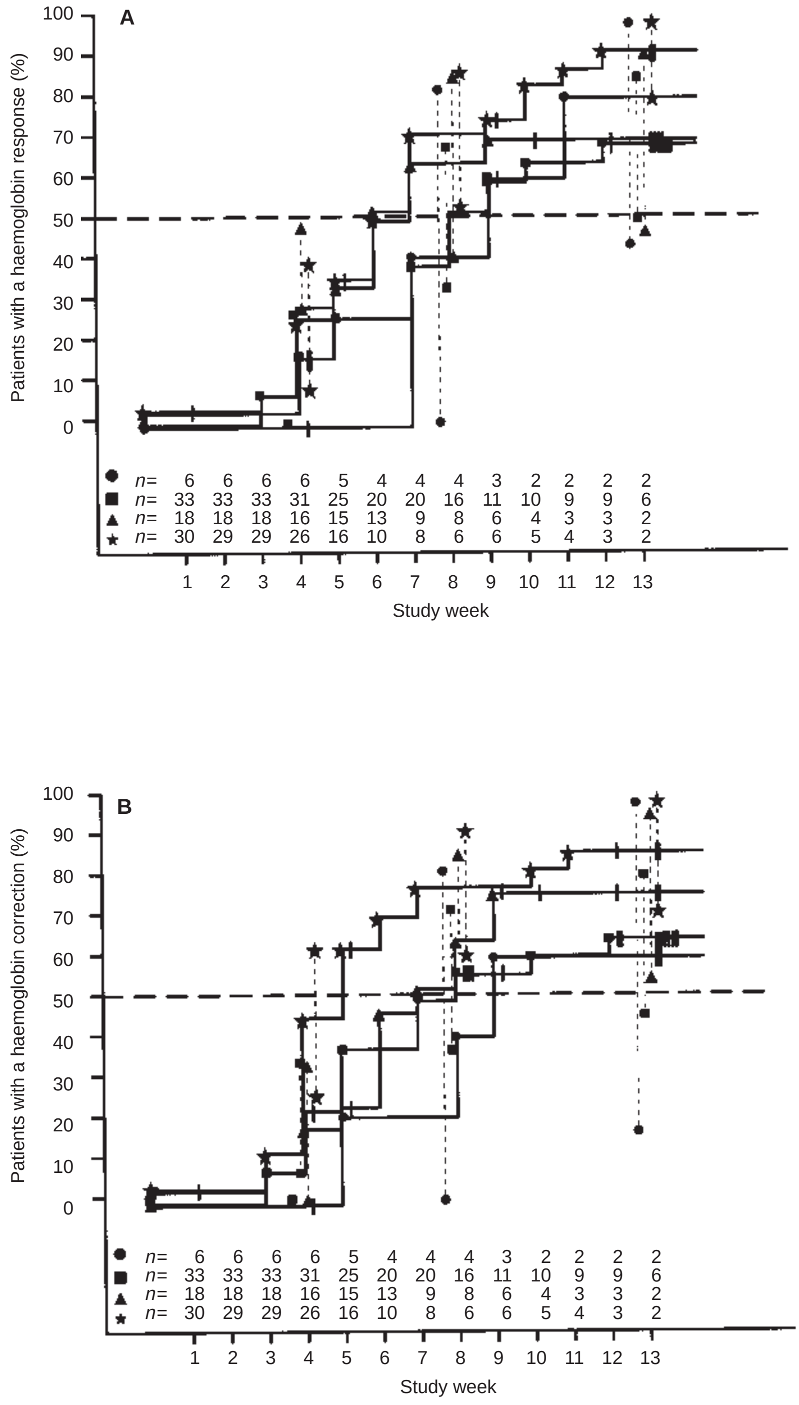

**Figure 2** Kaplan–Meier estimate of the cumulative percent of patients responding (panel **A**) and correcting (panel **B**) over study weeks. The NESP dose cohorts studied were 0.5 (●), 1.0(■), 2.25 (▲), and 4.5 (★) mcg $kg^{-1}$ $wk^{-1}$. The *n*'s represent the number of patients who have yet to achieve the endpoint or become censored at the start of each study week. An 'I' on the plot indicates a censored observation. The vertical dashed lines at weeks 4, 8 and 13 are 95% confidence intervals of the cumulative percents at these time points

(95% confidence interval) increased to 1.71 (0.91, 2.52), 2.63 (1.54, 3.71) and 2.91 (2.17, 3.65).

Similarly, the incidence of red blood cell transfusions from week 5 to the end of treatment also suggests a dose–response relationship. The transfusion rates (95% confidence interval) for patients receiving at least one dose of study drug were 24% (11%, 42%), 17% (4%, 41%) and 7% (1%, 22%) in the NESP 1.0, 2.25 and 4.5 mcg $kg^{-1}$ $wk^{-1}$ cohorts, respectively.

We also investigated the impact of dose on the time required for response. Figure 2 illustrates the relationship between parameters of efficacy over time. A consistent trend is observed with respect to the rapidity of achieving the desired endpoints of response

(panel A) or correction (panel B). The median time to achieving any of these endpoints was shortest with the higher doses.

### Quality of life findings

The relationship between improvements in haemoglobin and improvement in subject self-reported fatigue, as measured by the FACT-Fatigue scale, is shown in Table 3. The difference between patients whose haemoglobin decreased and patients whose haemoglobin increased by > 2.0 g $dl^{-1}$ was 14.2 points on the FACT-Fatigue scale.

### DISCUSSION

Chronic anaemia of cancer is a common clinical problem for which no treatment is currently approved other than red blood cell transfusions. RHuEPO has been used off-label in this setting, but the published data are not clear with respect to a dose–response relationship for rHuEPO in this setting. In addition, because of the chronic nature of this condition, attributes consistent with less frequent administration would be desirable. In recent studies, NESP has been shown to have an increased terminal half-life and the ability to ameliorate anaemia in cancer patients undergoing multiple cycles of chemotherapy. The current study was undertaken to explore the safety and efficacy of multiple doses of NESP in patients not receiving chemotherapy who have chronic anaemia of cancer.

In this study, NESP was safe and well tolerated at doses from 0.5 to 4.5 mcg $kg^{-1}$ $wk^{-1}$ when administered weekly by subcutaneous injection. The safety profile for NESP is generally similar to that reported for rHuEPO with adverse events predominantly due to the underlying medical condition of patients with cancer.

In patients with chronic anaemia of cancer, at all doses tested, NESP demonstrated clinical activity, with apparent dose–response relationships, for all efficacy parameters. NESP, when administered once-weekly over a 12-week period, increased haemoglobin values ≥ 2.0 g $dl^{-1}$ over baseline in ≥ 60% of all patients treated at each dose tested. These rates are comparable to the 62% haemoglobin response rate (Ludwig et al, 1995) and the 63.5% haematocrit response rate (Abels et al, 1991: Ludwig et al, 1994) reported in a similar patient population after 12 weeks of dosing three times weekly with rHuEPO (100 to 150 U $kg^{-1}$). The higher doses (2.25 and 4.5 mcg $kg^{-1}$ $wk^{-1}$) of NESP tested in this study appear to produce a higher response rate in this group of patients than the rate that has been historically reported. This is likely due to a robust dose–response relationship of erythropoietic agents in this setting. The lack of a control group in this study, however, limits the ability to draw firm conclusions in this regard.

Anaemia has been shown to be a major factor in the fatigue and decreased quality of life observed in cancer patients. Ameliorating anaemia with its attendant morbidity more rapidly is a desired goal of therapy. Importantly, our time to response or correction analysis suggests that higher dosing with NESP early in the course of treatment may result in a more rapid response. This is a unique observation and, if confirmed in future studies, has obvious implications for the use of this agent in patients who are significantly impaired by fatigue.

This study has limitations. The lack of a control group makes it difficult to compare the results of this study with others in the literature using rHuEPO as the treatment. In addition, it cannot be firmly concluded that the results seen in this study are different from what would be observed with placebo therapy. However, this seems unlikely given the dose-dependent relationships that were observed for most measures of efficacy and given the high rates of response seen in this study compared with what would be expected from historical controls. The open-label nature of this study calls into question the results observed in the self-reported health-related quality of life measurements. However, this portion of the study was intended primarily to assess the feasibility and timing of measuring health-related quality of life in this patient population. Finally, the weekly administration of NESP should be compared with less frequent dosing, a schedule that seems plausible given the high response rates observed in this study. These results suggest that more rigorous, placebo-controlled studies that explore longer dose intervals and confirm the dose–response and time to response findings are warranted.

### ACKNOWLEDGEMENTS

Janet Lee Nichol, MS; Suzana Giffin, PharmD; Joel Kallich, PhD; and MaryAnn Foote, PhD, assisted in the writing of this manuscript.

### REFERENCES

Abels RI, Larholt KM, Krantz KD and Bryant EC (1991) Recombinant human erythropoietin (r-HuEPO) for the treatment of anemia of cancer. In: *Blood Cell Growth Factors: Their Present and Future use in Hematology and Oncology. Proceedings of the Beijing Symposium*. Murphy MJ Jr (ed.). AlphaMed Press: Dayton, OH, 121–142

Cella D (1997) The Functional Assessment of Cancer Therapy-Anemia (FACT-An) Scale: a new tool for the assessment of outcomes in cancer anemia and fatigue. *Semin Hematol* **34**: 13–19

Cooke K, Stoney G, Pistillo J, Del Castillo J, Molineux G and Coccia MA (2000) Anemia of chronic disease (ACD) in a rodent model is similar to human ACD and can be alleviated by ARANESP™ treatment. *Blood* **96**: 8a (abstract 20)

Egrie JC, Dwyer E, Lykos M, Hitz A and Browne JK (1997) Novel erythropoiesis stimulating protein (NESP) has a longer serum half-life and greater in vivo biological activity than recombinant human erythropoietin (rHuEPO). *Blood* **90**: 56a–57a (abstract 243)

Glaspy J, Colowick AB and Heatherington A (2000) Novel erythropoiesis stimulating protein (NESP) exhibits a prolonged serum half-life ($T_{1/2}$) in oncology patients (pts). *Proc Am Soc Clin Oncol* **19**: 54a (abstract 210)

Henry DH and Abels RI (1994) Recombinant human erythropoietin in the treatment of cancer and chemotherapy-induced anemia: results of double-blind and open-label follow-up studies. *Semin Oncol* **21**: 21–28

Jelkmann W (1998) Proinflammatory cytokines lowering erythropoietin production. *J Interferon Cytokine Res* **18**: 555–559

Ludwig H and Fritz E (1998) Anemia in cancer patients. *Semin Oncol* **25**: 2–6

Ludwig H, Fritz E, Leitgeb C, Pecherstorfer M, Samonigg H and Schuster J (1994) Prediction of response to erythropoietin treatment in chronic anemia of cancer. *Blood* **84**: 1056–1063

Ludwig H, Sundal E, Pecherstorfer M, Leitgeb C, Bauernhofer T, Beinhauer A, Samonigg H, Kappeler AW and Fritz E (1995) Recombinant human erythropoietin for the correction of cancer associated anemia with and without cytotoxic chemotherapy. *Cancer* **76**: 2319–2329

Macdougall IC, Gray SJ, Elston O, Breen C, Jenkins B, Browne J and Egrie J (1999) Pharmacokinetics of novel erythropoiesis stimulating protein compared with Epoetin alfa in dialysis patients. *J Am Soc Nephrol* **10**: 2392–2395

Miller CB, Jones RJ, Piantadosi S, Abeloff MD and Spivak JL (1990) Decreased erythropoietin response in patients with anemia of cancer. *N Engl J Med* **322**: 1689–1692

Ozguroglu M, Arun B, Demir G, Demirelli F, Mandel NM, Buyukunal E, Serdengecti S and Berkarda B (2000) Serum erythropoietin level in anemic cancer patients. *Med Oncol* **17**: 29–34

© 2001 Cancer Research Campaign
